# Analysis of risk factors for deep vein thrombosis after spinal infection surgery and construction of a nomogram preoperative prediction model

**DOI:** 10.3389/fcimb.2023.1220456

**Published:** 2023-08-03

**Authors:** Dongcheng Xu, Xiaojiang Hu, Hongqi Zhang, Qile Gao, Chaofeng Guo, Shaohua Liu, Bo Tang, Guang Zhang, Chengran Zhang, Mingxing Tang

**Affiliations:** ^1^ Department of Spine Surgery and Orthopaedics, Xiangya Hospital, Central South University, Changsha, China; ^2^ China for Geriatric Disorders, Xiangya Hospital, Central South University, Changsha, China

**Keywords:** spine, infection, deep vein thrombosis, risk factors, predictive model, LASSO, nomogram, mNGS (metagenomic next-generation sequencing)

## Abstract

**Objective:**

To investigate the differences in postoperative deep venous thrombosis (DVT) between patients with spinal infection and those with non-infected spinal disease; to construct a clinical prediction model using patients’ preoperative clinical information and routine laboratory indicators to predict the likelihood of DVT after surgery.

**Method:**

According to the inclusion criteria, 314 cases of spinal infection (SINF) and 314 cases of non-infected spinal disease (NSINF) were collected from January 1, 2016 to December 31, 2021 at Xiangya Hospital, Central South University, and the differences between the two groups in terms of postoperative DVT were analyzed by chi-square test. The spinal infection cases were divided into a thrombotic group (DVT) and a non-thrombotic group (NDVT) according to whether they developed DVT after surgery. Pre-operative clinical information and routine laboratory indicators of patients in the DVT and NDVT groups were used to compare the differences between groups for each variable, and variables with predictive significance were screened out by least absolute shrinkage and operator selection (LASSO) regression analysis, and a predictive model and nomogram of postoperative DVT was established using multi-factor logistic regression, with a Hosmer- Lemeshow goodness-of-fit test was used to plot the calibration curve of the model, and the predictive effect of the model was evaluated by the area under the ROC curve (AUC).

**Result:**

The incidence of postoperative DVT in patients with spinal infection was 28%, significantly higher than 16% in the NSINF group, and statistically different from the NSINF group (P < 0.000). Five predictor variables for postoperative DVT in patients with spinal infection were screened by LASSO regression, and plotted as a nomogram. Calibration curves showed that the model was a good fit. The AUC of the predicted model was 0.8457 in the training cohort and 0.7917 in the validation cohort.

**Conclusion:**

In this study, a nomogram prediction model was developed for predicting postoperative DVT in patients with spinal infection. The nomogram included five preoperative predictor variables, which would effectively predict the likelihood of DVT after spinal infection and may have greater clinical value for the treatment and prevention of postoperative DVT.

## 1 Introduction

Spinal infection is a rare disease. Its incidence rate is 0.001%~0.004% and its estimated mortality rate ranges between 2 and 4% (Duarte and Vaccaro, 2013). In recent years, the incidence rate and mortality rate of spinal infection is on the rise (Babic and Simpfendorfer, 2017; Sato et al., 2019). The main reason of spinal infection is the colonization of pathogens in the spine, and its pathogens mainly include various bacteria and viruses. Diagnosis of spinal infection is mainly based on clinical symptoms and signs, laboratory tests and imaging examinations. Routine microbial culture as direct evidence for infection diagnosis is the gold standard for diagnosis of spinal infection (Urrutia and Bono, 2022). Antibiotics are the main treatment for spinal infection. Surgical treatment should be used for patients with severe illness and no significant improvement (Herren et al., 2017; Scheyerer et al., 2022).

Deep venous thrombosis (DVT) is a well-known and feared surgical complication, as well as a leading cause of death. The incidence of venous thrombosis following spinal surgery varies between 0.31% and 31%. DVT may result in substantial morbidity, poor quality of life, and even death. It may also lead to increased medical costs and a considerable financial burden on individuals and their families (Wang and Wu, 2022). Studies have showed that risk factors for venous thrombosis following spinal surgery are associated with high age, female, spinal fusion, big volume blood loss patients, operation time, and hypertension, diabetes, and walking issue. A series of risk prediction models for deep venous thrombosis after surgery for patients with spinal surgery and fracture (Cheng et al., 2022; Hu et al., 2022; Yan et al., 2022).

Recently, the association between infection and venous thrombosis has long been recognized (Colling et al., 2021). Systemic or localized infections increase the risk of thrombosis about 2~20 times and are independent risk factors for thromboembolic diseases (Beristain-Covarrubias et al., 2019). Neutrophils, monocytes, and platelets interact with each other and the endothelium in host defense and also play critical roles in the formation of venous thromboembolism (Beristain-Covarrubias et al., 2019; Colling et al., 2021). However, clinical research on thrombosis after spinal infection surgery has not received much attention yet. It is generally believed that the incidence of thrombosis should be higher than that of a single spinal surgery or infection. Study has showed that patients with spine infections requiring irrigation and débridement may be at considerably increased risk for DVT. Lambrechts MJ et al. found that 14.3% of patients undergoing spine irrigation and débridement with subsequent peripherally inserted central catheter (PICC) placement developed postoperative DVT (Lambrechts et al., 2022). In this study, we compared the incidence of DVT after surgery for spinal infection, analysed the risk factors for DVT after surgery for spinal infection, screened the predictor variables by LASSO regression and developed a predictive model for the prediction of postoperative DVT in patients with spinal infection.

## 2 Method

### 2.1 Study subjects

314 cases of spinal infection from January 2016 to December 2021 were collected from Xiangya Hospital of Central South University; 314 cases of non-infected spinal disease from January 2016 to December 2021 were randomly selected from Xiangya Hospital of Central South University. The spinal infection cases included 126 cases tested by metagenomic next-generation sequencing (mNGS). Ethical approval: Institutional ethics review board approval was obtained (IRB#: 201303232). This study was approved by the Ethics Committee of Xiangya Hospital Central South University.

#### 2.1.1 Inclusion criteria

Inclusion criteria for spinal infection cases: (1) complete clinical information, including gender, age and temperature; (2) routine preoperative laboratory indices; (3) patients treated surgically; (4) lesion specimens identified as pathogenic microbial infections by pathological examination, bacterial culture and staining, DNA testing of Mycobacterium tuberculosis flora and macrogenome sequencing; (5) postoperative vascular ultrasound results of both lower limbs.

Inclusion criteria for non-infected spinal disease cases: (1) complete clinical information, including gender and age; (2) patients were treated surgically; (3) patients did not have infectious diseases or signs of infection (e.g. fever, elevated blood count, etc.); (4) postoperative vascular ultrasound findings in both lower limbs.

#### 2.1.2 Exclusion criteria

Patients meeting the following criteria will be excluded: (1) patients with infections elsewhere in the body; (2) patients with previous bleeding disorders (e.g. haemophilia, thrombocytopenic purpura, etc.) or vascular disorders (e.g. varicose veins, thrombophlebitis, etc.) (Boender et al., 2016; Chang et al., 2018); (3)Patients treated with preoperative anticoagulation or antiplatelet drugs; (4) patients with preoperative tests suggestive of DVT; (5) patients with critical multi-organ failure; (6) patients with malignancies (Falanga et al., 2013; Falanga et al., 2015).

### 2.2 Research methods

#### 2.2.1 Pathogenic microbial detection methods

One of the following diagnostic criteria can be met: (1) pathological examination of the spinal lesion suggests a spinal infection (including inflammatory lesions, septicemia, etc.); (2) bacterial culture or staining of the spinal lesion detects bacteria or fungi (Berbari et al., 2015); (3) mNGS of the spinal lesion (Xu et al., 2022): high quality sequences are screened by FastQC software, removing sequences with connectors, low quality bases and too short (<50bp) sequences. BowtiE2 was used for inter-sequence comparison and to remove host-associated reads. The processed sequences were compared with the Guangzhou Sage pathogenic microbial database for BWA analysis to obtain the number of detected sequences of pathogenic microorganisms; (4) positive spinal lesions for Mycobacterium tuberculosis cluster DNA detection (Xpert MTB/RIF) (Dorman et al., 2018).

#### 2.2.2 Routine laboratory test result data collection

Patient’s preoperative platelet (PLT), plateletcrit (PCT), prothrombin time (PT), activeated partial thromboplastin time (APTT), international normalized ratio (INR), D-dimer (DD), white blood cell (WBC), red blood cell (RBC), hemoglobin (HGB), neutrophil (Neut), lymphocyte (Lymph), eosinophil (EO), basophil (BASO), monocyte (Mono), neutrophil% (Neut%), lymphocyte% (Lymph%), basophil% (BASO%), eosinophil% (EO%), monocyte% (Mono%), red blood cell distribution width (RDW), mean platelet volume (MPV), total protein (TP), albumin (A), globulin (G), albumin to globulin ratio (A/G), alanine transaminase (ALT), aspartate transaminase (AST), blood urea nitrogen (BUN), creatinine (Cr), triglyceride (TG), cholesterol (Chol), high density lipoprotein (HDL), low density lipoprotein (LDL), glucose (GLU), erythrocyte sedimentation rate (ESR), C-reactive protein (CRP), procalcitonin (PCT).

#### 2.2.3 DVT diagnostic criteria

The diagnosis was confirmed on the basis of postoperative vascular ultrasound findings in both lower limbs (Lim et al., 2018).

### 2.3 Statistical methods

Measures that conformed to a normal distribution were described using the mean ± standard deviation and t-tests for two independent samples were used for comparisons between groups. Measures that did not conform to a normal distribution were described using the median and percentile, and comparisons between groups were made using a non-parametric test. The chi-square test was used for inter-group comparisons of the count data. Predictors were screened using the least absolute shrinkage and operator selection (LASSO) regression technique, and postoperative D-dimer prediction models and column line plots (nomograms) were developed using multifactorial logistic regression. The calibration curve of the model was plotted using the Hosmer-Lemeshow goodness-of-fit test. The predictive effect of the model was evaluated by calculating the area under the ROC curve (AUC). 60% of spinal infection cases were randomly selected as the training set and the remaining spinal infection cases were included in the validation set for internal validation of the prediction model. Statistical analyses and image plotting were performed using R software (version 4.2.2; R Foundation for Statistical Computing, Vienna, Austria) and differences were considered statistically significant when p<0.05.

## 3 Result

A total of 314 cases of all patients with spinal infection (SINF) and 314 cases of randomly selected patients with non-infected spinal disease (NSINF) who met the inclusion criteria and were admitted to Xiangya Hospital of Central South University from 1 January 2016 to 31 December 2021 were included in the study. The general data of patients in the SINF and NSINF groups are analysed in **Table 1**. There was no statistical difference in age and gender between the SINF and NSINF, The results of the chi-square test for postoperative DVT between the SINF and NSINF groups are shown in **Table 2**. There was a significant difference in the incidence of postoperative DVT between the two groups (p < 0.000), and the incidence of postoperative DVT was significantly higher in SINF than in NSINF.

**Table 1:** Characteristics of the clinical data of each group of patients.

Variables	SINF (n=314)	NSINF (n=314)	p value
Age, years	55 (46.25, 64)	54 (46, 63)	0.453
Gender, n (%)			0.936
female	132 (42)	133 (42)	
male	182 (58)	181 (58)	

**Table 2:** Results of the chi-square test analysis of postoperative DVT for each group of patients.

Group	DVT, n (%)		Total	χ^2^	p value
	positive	negative			
SINF group	89 (28)	225 (72)	314	14.053	< 0.000
NSINF group	50 (16)	264 (84)	314		
Total	139 (22)	489 (78)	628		

The SINF consisted of 126 cases tested by mNGS, including 110 bacterial infections (including 28 tuberculosis infections and 9 brucellosis infections), 6 fungal infections, 5 viral infections, 2 rickettsial infections, 1 mycoplasma infection and 2 undetected cases. See **Supplementary Information** for specific microbial species.

**Figure 1:**
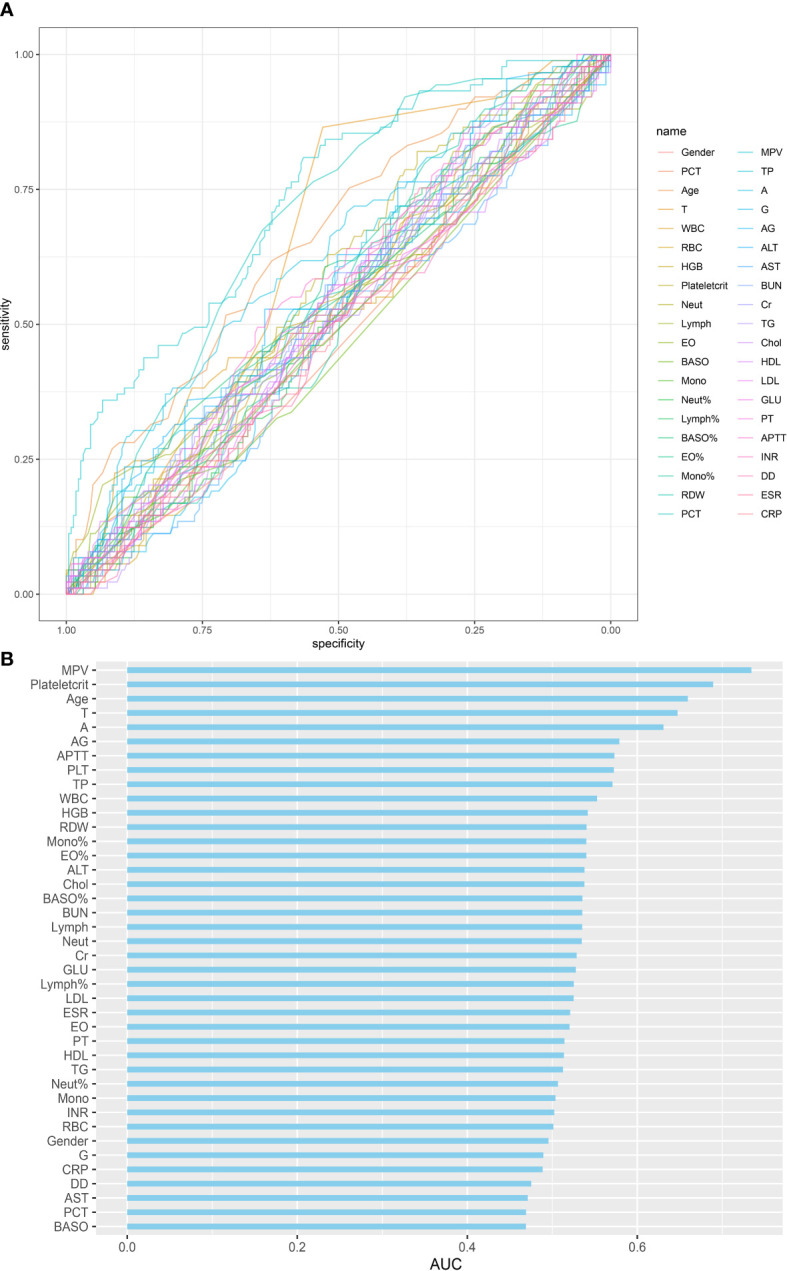
Single variables on DVT predictive capability: **(A)** ROC curve of single variables on DVT prediction; **(B)** AUC of single variables for DVT prediction.

The SINF cases were divided into a thrombotic group (DVT) and a non-thrombotic group (NDVT) based on postoperative lower limb vascular ultrasound findings. There were 89 patients in the DVT and 225 patients in the NDVT. The general data and each preoperative serological index of the two groups are shown in **Table 3**. The two groups differed in Age, T, PCT, Plateletcrit, MPV, A, AG and APTT (p<0.05). In this study, univariate ROC curves were plotted by these variables (**Figure 1A**) and their AUC results were plotted (**Figure 1B**).

**Table 3:** Univariate analysis of patients and variables for spinal infection cases.

Variables	Total (n = 314)	NDVT (n = 225)	DVT (n = 89)	p value
Gender, n (%)				0.983
female	132 (42)	94 (42)	38 (43)	
male	182 (58)	131 (58)	51 (57)	
Age, years	55 (46.25, 64)	53 (44, 63)	60 (53, 69)	< 0.001
T, °C	36.6 (36.5, 36.8)	36.5 (36.5, 36.8)	36.6 (36.6, 36.9)	< 0.001
WBC, 10^9/L	6.25 (4.8, 7.7)	6.3 (5, 7.7)	6.2 (4.5, 7.7)	0.146
RBC, 10^12/L	4.07 ± 0.6	4.08 ± 0.63	4.06 ± 0.51	0.836
HGB, g/L	117 (106.25, 132)	117 (105, 132)	119 (108, 133)	0.249
PLT, 10^9/L	241 (188.25, 310.75)	253 (190, 323)	225 (184, 277)	0.046
Neut, 10^9/L	4 (3, 5.4)	4 (3, 5.5)	3.8 (2.9, 5)	0.339
Lymph, 10^9/L	1.31 (1, 1.79)	1.4 (1, 1.7)	1.3 (0.9, 1.8)	0.332
EO, 10^9/L	0.1 (0.08, 0.2)	0.1 (0.08, 0.2)	0.1 (0.1, 0.2)	0.566
BASO, 10^9/L	0 (0, 0.03)	0 (0, 0.03)	0 (0, 0.02)	0.328
Mono, 10^9/L	0.5 (0.4, 0.7)	0.5 (0.4, 0.7)	0.5 (0.4, 0.7)	0.917
Neut%, %	65.85 (58.82, 72.1)	66 (58.3, 72.5)	65.6 (60.5, 70.9)	0.855
Lymph%, %	21.65 (16.72, 27.6)	21.8 (16.8, 27.9)	21.5 (16.7, 26.3)	0.486
BASO%, %	0.5 (0.3, 0.7)	0.5 (0.3, 0.6)	0.5 (0.3, 0.7)	0.325
EO%, %	1.8 (0.9, 3.2)	1.7 (0.9, 3.1)	2 (1.1, 3.3)	0.27
Mono%, %	8.8 (7.2, 10.5)	8.8 (6.8, 10.5)	8.8 (7.6, 10.3)	0.27
RDW, %	13.8 (12.9, 14.8)	13.8 (12.9, 14.9)	14.1 (13.1, 14.7)	0.266
Plateletcrit^*^, %	0.2 (0.16, 0.26)	0.22 (0.17, 0.28)	0.17 (0.14, 0.2)	< 0.001
MPV, fL	8.35 (7.52, 9.5)	8.71 (7.76, 9.9)	7.81 (6.8, 8.35)	< 0.001
TP, g/L	69.83 ± 6.72	70.26 ± 6.77	68.73 ± 6.52	0.066
A, g/L	36.86 ± 4.47	37.43 ± 4.48	35.4 ± 4.12	< 0.001
G, g/L	32.4 (28.6, 36.9)	32.7 (28, 36.9)	31.8 (29.3, 36.9)	0.773
A/G	1.15 (0.97, 1.31)	1.2 (1, 1.4)	1.07 (0.91, 1.29)	0.029
ALT, U/L	17.8 (11.4, 28.25)	17.9 (11.9, 28.6)	17.3 (10.3, 27.7)	0.296
AST, U/L	21.1 (16.8, 29.7)	21 (17.1, 30.3)	21.6 (16.5, 27)	0.424
BUN, mmol/L	4.85 (3.81, 6.12)	4.68 (3.81, 5.98)	5 (3.82, 6.35)	0.333
Cr, mmol/L	72 (61, 82.2)	72 (61, 82.2)	72.4 (62, 82.2)	0.430
TG, mmol/L	1.23 (0.88, 1.65)	1.23 (0.86, 1.68)	1.25 (0.95, 1.62)	0.729
Chol, mmol/L	4.26 (3.6, 5.07)	4.21 (3.58, 4.94)	4.36 (3.7, 5.14)	0.299
HDL, mmol/L	1.02 (0.86, 1.21)	1.02 (0.87, 1.2)	1.01 (0.85, 1.23)	0.706
LDL, mmol/L	2.68 (2.25, 3.24)	2.67 (2.2, 3.25)	2.73 (2.32, 3.19)	0.491
GLU, mmol/L	5.19 (4.77, 5.8)	5.23 (4.74, 5.88)	5.16 (4.8, 5.61)	0.446
PT, s	12.8 (12, 13.7)	12.7 (12, 13.7)	12.9 (12, 13.7)	0.695
APTT, s	32.6 (29.2, 36.35)	32.9 (29.4, 36.9)	31.2 (28.7, 35.3)	0.044
INR	1.02 (0.96, 1.09)	1.02 (0.96, 1.09)	1.02 (0.96, 1.09)	0.951
DD, mg/L	0.3 (0.17, 0.54)	0.3 (0.17, 0.52)	0.29 (0.19, 0.57)	0.493
ESR, mm/h	70.5 (40.25, 103.75)	70 (38, 105)	71 (51, 102)	0.561
CRP, mg/L	17.3 (6.65, 41.88)	17.3 (6.57, 42.2)	17.5 (6.81, 37.4)	0.755
Procalcitonin^**^, n (%)				0.337
negative	226 (72)	158 (70)	68 (76)	
positive	88 (28)	67 (30)	21 (24)	

*Plateletcrit (PCT) and Procalcitonin (PCT) have the same abbreviation, so their full names are used in this study.

**The Procalcitonin is defined as a count data because there are multiple clinical assays with different normal values between methods.

**Figure 2:**
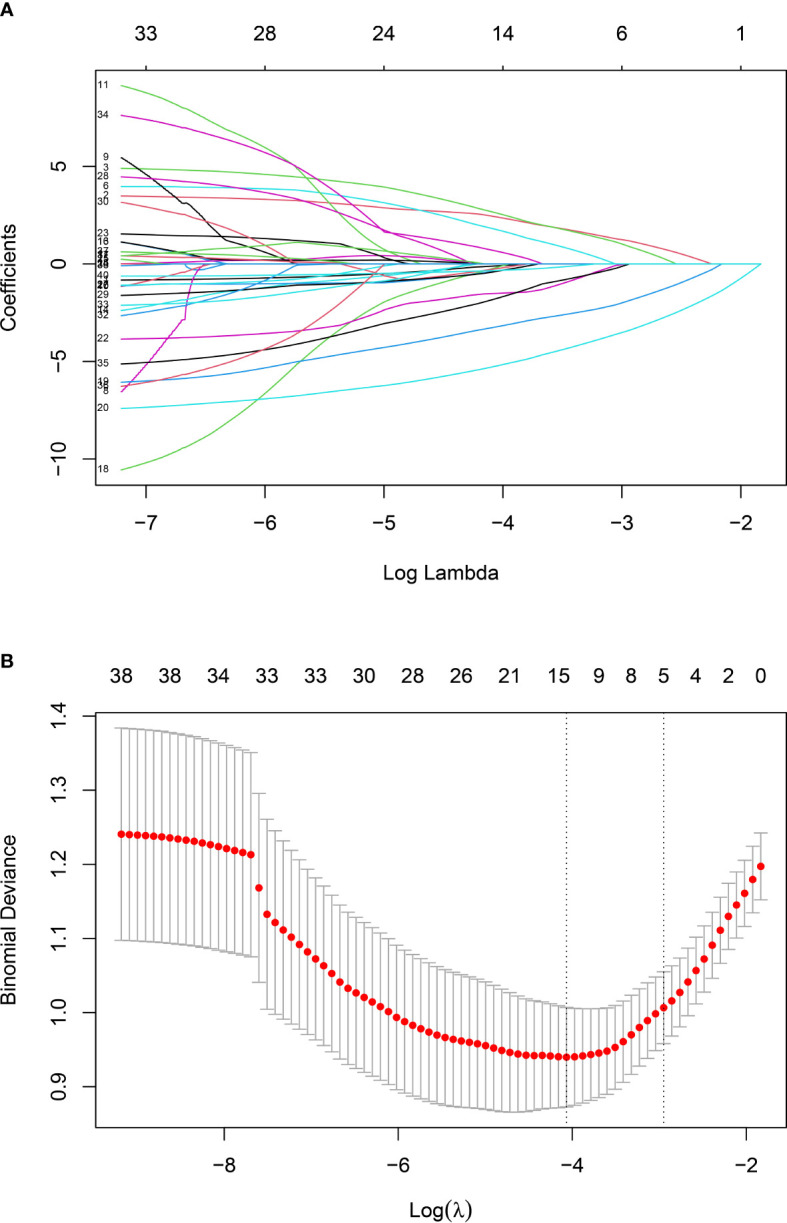
Screening variables by LASSO regression: **(A)** LASSO regression model graph. In this study, five non-zero coefficients were screened using Lambda.min as the criterion.; **(B)** Lambda (adjustment parameter) was obtained by cross-validated LASSO regression. The left dashed line is Lambda at minimum error (Lambda.min), the right dashed line is Lambda at standard error (Lambda.1-SE).

**Figure 3:**
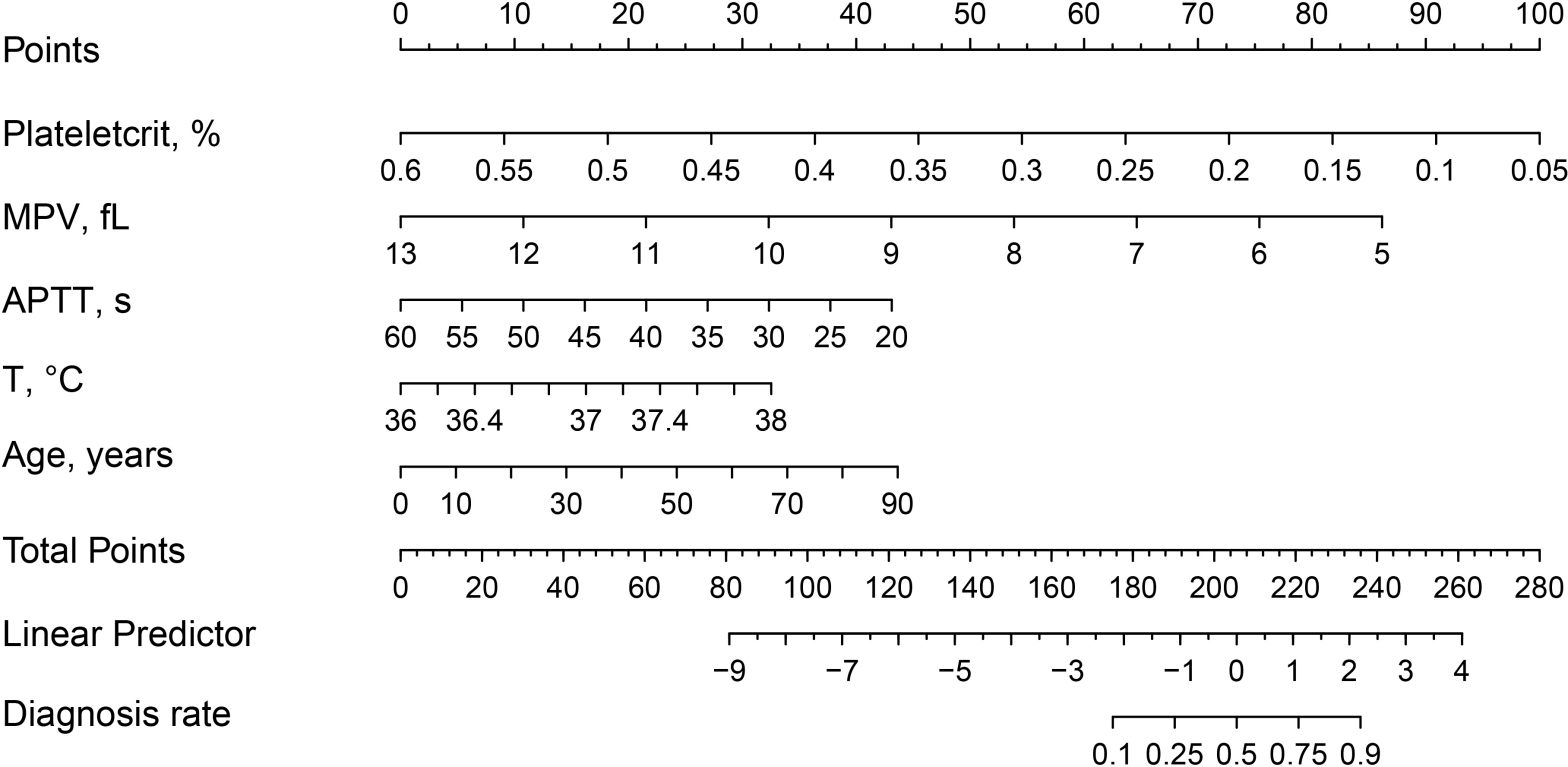
Nomogram and scoring method for postoperative DVT in patients with spinal infection. The corresponding score (top line) is found according to the value of each predictor variable (the line after each variable), then the values of the individual scores are summed to obtain the total score, and the corresponding predicted probability is based on the total score(bottom line).

In this study, five non-zero coefficient variables (**Figure 2**), including Plateletcrit, MPV, APTT, T and Age, were selected from 40 variables in patients with spinal infection by LASSO regression, and the corresponding postoperative DVT prediction model was developed by multifactorial logistic regression analysis based on these five variables. In this study, a nomogram of postoperative DVT in patients with spinal infection (**Figure 3**) was constructed based on the prediction model, and the results of the prediction model were presented visually to facilitate the preoperative assessment of patients with spinal infection.

**Figure 4:**
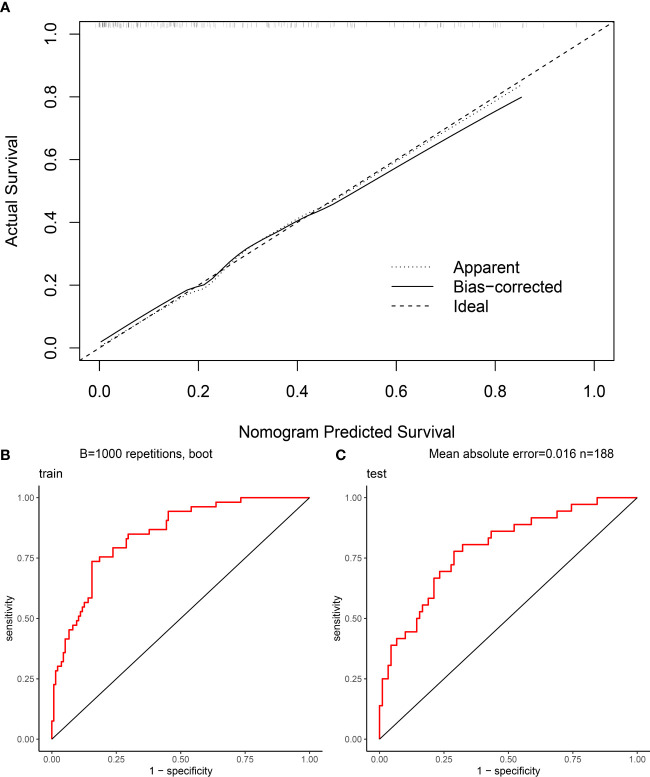
Evaluation of nomogram clinical prediction model: **(A)** Calibration curve of the model; **(B)** ROC curve of the model for the training cohort, and its AUC was 0.8457; **(C)** ROC curve of the model for the validation cohort, and its AUC was 0.7917.

The Hosmer-Lemeshow goodness of fit test (**Figure 4A**) shows a good fit between the nomogram predicted probabilities and the actual probabilities. This indicates that there is no deviation from a perfect fit between the predicted and observed values. In addition, this study plotted the ROC curve for the model, which had an AUC of 0.8457 (**Figure 4B**) in the training cohort and 0.7917 (**Figure 4C**) in the validation cohort.

## 4 Discussion

In this study, a clinical prediction model for postoperative DVT in patients with spinal infection was developed using LASSO regression and visualised by nomogram. The nomogram had five preoperative predictor variables including Plateletcrit, MPV, APTT, T and Age. The goodness of fit test shows a good fit between the nomogram predicted probabilities and the actual probabilities. In addition, the ROC curve of the model was plotted in this study. Its AUC was 0.8457 in the training cohort and 0.7917 in the validation cohort. The prediction model nomogram showed good preoperative predictive ability and clinical value.

In recent years, with the progression of an ageing population and the misuse of antibiotics, there has been an upward trend in the incidence of spinal infections (Babic and Simpfendorfer, 2017; Dunn and Ben Husien, 2018), particularly with various drug-resistant bacterial infections, leading to an increased probability of serious complications. Patients with spinal infections who experience spinal bone destruction often experience skeletal related events (SREs) such as severe bone pain, pathological fractures, spinal cord compression and hypercalcaemia. Surgical treatment is usually required in this case. DVT is one of the most common complications of surgery and one of the most common causes of death in surgical patients (Blitzer and Eisenstein, 2021). Numerous studies have shown that the incidence of postoperative venous thromboembolism is significantly higher in surgical patients than preoperatively (Zhang et al., 2021; Jiang et al., 2022). Patients undergoing spinal surgery are more likely to develop postoperative DVT due to the need for prolonged bed rest after surgery, as evidenced by the study by Hengyan Zhang et al. (2021). The occurrence of postoperative DVT in patients has been associated with several preoperative factors, such as advanced age and infection. Studies have shown that infection is an important factor influencing the development of DVT, and that the dysfunction of haemostasis and vascular barrier dysfunction caused by infection promotes thrombosis (Colling et al., 2021). In this study, 314 SINF and 314 NSINF cases were included. There were no significant differences between SINF and NSINF in terms of age and gender, while there were statistically significant differences in terms of postoperative DVT. Therefore, for patients with spinal infection, the likelihood of postoperative DVT would be significantly increased.

This study included 126 cases that were tested for mNGS. The 2018 guidelines of the Infectious Diseases Society of America and the American Society for Microbiology continue to use microbiological culture of spinal lesions as a criterion for laboratory diagnosis of spinal infections (Miller et al., 2018). However, studies have shown that positive microbial cultures of spinal infection lesions are low (Zhang et al., 2023), making it difficult to meet clinical diagnostic needs, and the long culture period for some microorganisms (e.g. Mycobacterium tuberculosis) may lead to delays in treatment. mNGS is extremely sensitive and specific for the detection of pathogenic microorganisms (Zhang et al., 2023), and with advances in technology, a rapid 50-minute median time mNGS assays (Gu et al., 2021). Therefore, mNGS may become an effective clinical tool for accurate and rapid diagnosis of infections.

In this study, a total of 314 patients with spinal infections were included. They were divided into 89 cases of DVT and 225 cases of NDVT according to the results of the vascular ultrasound of both lower limbs. univariate analysis of 40 preoperative data from both groups showed significant differences in Age, T, PLT, Plateletcrit, MPV, A, AG and APTT. By plotting ROC curves and calculating AUC, we found that all individual variables did not show significant value for predicting DVT (AUC < 0.75), suggesting that a single variable is difficult to accurately predict the likelihood of postoperative DVT. Therefore, to more accurately predict postoperative DVT, this study used LASSO regression to screen out five variables, including Plateletcrit, MPV, APTT, T and Age, and construct a DVT prediction model by logistic regression. The model achieved an AUC of 0.8457 in the training cohort and 0.7917 in the validation cohort, indicating that the model has relatively good predictive value. The fitted curves also showed a good fit. This study found that a decrease in preoperative Plateletcrit, MPV and APTT levels predicted an increased likelihood of DVT in patients postoperatively, while an increase in T and Age predicted an increased likelihood of DVT in patients postoperatively.

Marina Panova-Noeva et al. observed that MPV was lower in cases where thrombosis occurred compared to controls (Panova-Noeva et al., 2020), a phenomenon that has been validated in other studies on thrombosis (Lippi et al., 2016; Braester et al., 2021). In infectious diseases, MPV levels can help to indicate the onset of inflammation. For example, in cases of acute appendicitis, MPV levels were significantly lower than in controls (Fan et al., 2017). Plateletcrit, which is the percentage of platelet volume in peripheral blood to whole blood volume, is the product of PLT and MPV, and decreased levels of Plateletcrit have been suggested to be associated with sepsis and poor prognosis in systemic inflammatory diseases in established studies (Sayed et al., 2020; Yardımcı et al., 2021). Shortening of APTT as an indicator of coagulation is usually indicative of a hypercoagulable state, and APTT light transmission waveform analysis is currently used by some investigators as a tool to detect infection and assess its prognosis (Chopin et al., 2006). The correlation between fever and infection has been well documented (Lu et al., 2015), and clinically, in non-serious patients, an increase in temperature is also often indicative of the onset or exacerbation of infection. A clinical trial has shown that fever is common in patients with DVT and has a worse prognosis (Barba et al., 2011). Advanced age is known to be an independent risk factor for thrombosis (Abad Rico et al., 2010). Recent studies have also shown that age has shown greater value in the diagnosis and prediction of DVT. Kelly Broen et al. used age-adjusted DD, and the adjusted DD threshold showed higher specificity and negative predictive value for the exclusion diagnosis of DVT (Broen et al., 2016). Jian Xiang Wu et al. used a similar approach and used age-adjusted-DD for preoperative DVT screening (Wu et al., 2021). Advanced age is also a risk factor for various infectious diseases, and studies have shown that the risk of urinary tract and surgical site infections will increase with age (Cheadle, 2006; Tandogdu and Wagenlehner, 2016). The morbidity and mortality of sepsis also increases significantly with age (Kingren et al., 2021). A large number of previous studies as well as clinical practice have shown that DD is highly sensitive for the diagnosis of DVT exclusion and guidelines published by the American Society of Hematology have identified DD as a preferred screening indicator for thrombosis (Lim et al., 2018). However, in the present study, we observed that preoperative DD levels did not show good predictive value for postoperative DVT in patients with spinal infections. We speculate that this may be related to the short half-life of DD. However, whether this phenomenon exists in other diseases still needs to be verified by more studies.

In the existing study, we noted that Xin Yan et al. predicted the risk of postoperative DVT progression in patients undergoing spinal surgery by plotting a nomogram, and they eventually screened five preoperative and intraoperative indicators, which included the time of surgery (Yan et al., 2022). Unlike their study, firstly, the present study included a large number of cases for calculation in patients with spinal infection, making the nomogram more relevant. Secondly, the present study included more routine preoperative serological indicators, which made it easier for clinicians to assess. Of course, there are differences in the outcomes we predict, with the present study predicting the occurrence rather than progression of DVT. Kimon Bekelis et al. analysed several risk factors for postoperative complications of spinal surgery, including DVT, and presented them in the form of an odds ratio (OR) (Bekelis et al., 2014). However, they did not present the predictive model visually, and in this respect, nomogram showed a greater advantage. In addition, we note that several studies have explored new methods for the diagnosis and prediction of DVT. A pilot study has shown that patients with musculoskeletal tumors can be defined as hypercoagulable by preoperative thromboelastography. And patients with a preoperative defined hypercoagulable state are more likely to develop DVT postoperatively (Sabharwal et al., 2023). Andreas G Tsantes et al. analyzed preoperative and postoperative rotational thromboelastometry parameters in patients with malignant bone tumors. Their accuracy for postoperative thrombus prediction was found to be significantly better than that of DD (Tsantes et al., 2023). Another study on rotational thromboelastometry analyzed data from patients with hip fractures and found a number of abnormal rotational thromboelastometry abnormalities associated with the development of venous thromboembolism (VTE), with preoperative clot formation time showing good performance in detecting symptomatic VTE (Tsantes et al., 2021). A study on spinal fractures analyzed risk factors for preoperative DVT, which included DD, and the adjusted optimal threshold for DD was 1.08ug/ml (Ma et al., 2021).

In this study, clinical data were retrospectively collected from patients undergoing surgery for spinal infection in Hunan Province, but there are geographical differences in the incidence of spinal infection, for example, Mycobacterium tuberculosis infection may be related to regional economic level and climatic environment (European Centre for Disease Prevention and Control and WHO Regional Office for Europe, 2022). Therefore, the applicability of this prediction model to other regions still needs further validation. This is a retrospective study and due to the absence of some preoperative data, this study excluded some indicators, such as height and weight, which may have value for preoperative prediction of DVT. Therefore, we intend to conduct further prospective studies based on this study to improve the accuracy of preoperative prediction of DVT.

## 5 Conclusion

In this study, a nomogram was developed to predict postoperative DVT in patients with spinal infection. The nomogram includes five preoperative predictor variables, which will effectively predict the likelihood of DVT after surgery. This nomogram is valuable in predicting postoperative DVT and will help clinicians to decide whether early intervention is needed based on the preoperative prediction results and the patient’s specific situation.

## Data availability statement

The raw data supporting the conclusions of this article will be made available by the authors, without undue reservation.

## Ethics statement

The studies involving humans were approved by Ethics Committee of Xiangya Hospital Central South University. The studies were conducted in accordance with the local legislation and institutional requirements. The ethics committee/institutional review board waived the requirement of written informed consent for participation from the participants or the participants’ legal guardians/next of kin because All subjects signed a consent form.

## Author contributions

DX and MT designed research, performed research, analyzed data, and wrote the paper. XH, HZ, QG, CG and SL developed the idea for the study. XH, BT, GZ and CZ collected the data. All authors contributed to the article and approved the submitted version.
