# Supplementary material for: Analysis of risk factors for deep vein thrombosis after spinal infection surgery and construction of a nomogram preoperative prediction model
Source: Front Cell Infect Microbiol. 2023 Aug 3;13:1220456. doi: 10.3389/fcimb.2023.1220456 (PMC10435901; doi:10.3389/fcimb.2023.1220456)
Supplement: Supplementary file 1 [file Table_1.docx]

| Microbial species | Quantity |
| --- | --- |
| Epstein-Barr virus, EBV | 1 |
| Mycobacterium intracellulare | 1 |
| Coxiella burnetii | 2 |
| Staphylococcus epidermidis | 5 |
| Propionibacterium | 11 |
| Brucella | 9 |
| prevotella | 3 |
| Bacaeroides fragilis | 1 |
| Escherichia coli | 4 |
| Pseudomonas putida | 1 |
| Klebsiella pneumoniae | 2 |
| Bacteroides caccae | 1 |
| Citrobacter freudii | 3 |
| Streptococcus mitis | 1 |
| Rhodotorula mucilaginosa | 1 |
| mycobacterium tuberculosis | 28 |
| Ureaplasma urealyticum | 1 |
| Staphylococcus aureus | 12 |
| Candida parapsilosis | 2 |
| Fusobacterium nucleatum | 1 |
| oralis streptococci | 2 |
| Micrococcus | 2 |
| corynebacterium | 1 |
| Actinobacteria | 2 |
| neisseria mucosa | 1 |
| Aerococcus | 1 |
| Aspergillus | 1 |
| Human herpesvirus | 1 |
| Staphylococcus hominis | 1 |
| Brevibacterium casei | 1 |
| Citrobacter sedlakii | 1 |
| Stenotrophomonas maltophilia | 2 |
| Streptococcus thermophilus | 1 |
| Streptococcus dysgalactiae | 1 |
| Pseudomonas aeruginosa | 4 |
| Staphylococcus capitis | 3 |
| Aspergillus terreus | 1 |
| Torque teno virus | 3 |
| Burkholderia cepacia | 2 |
| Aspergillus fumigatus | 1 |
| Enterobacter cloacae | 1 |
| Bifidobacterium | 1 |
| Undetected | 2 |
